# Supplementary material for: A Global Screen for Assembly State Changes of the Mitotic Proteome by SEC-SWATH-MS
Source: Cell Syst. 2020 Feb 26;10(2):133–155.e6. doi: 10.1016/j.cels.2020.01.001 (PMC7042714; doi:10.1016/j.cels.2020.01.001)

P51812 | KS6A3_HUMAN | RPS6KA3 ISPK1 MAPKAPK1B RSK2
Monomer MW [kDa]: 83.736 Monomer expected elution fraction: 42

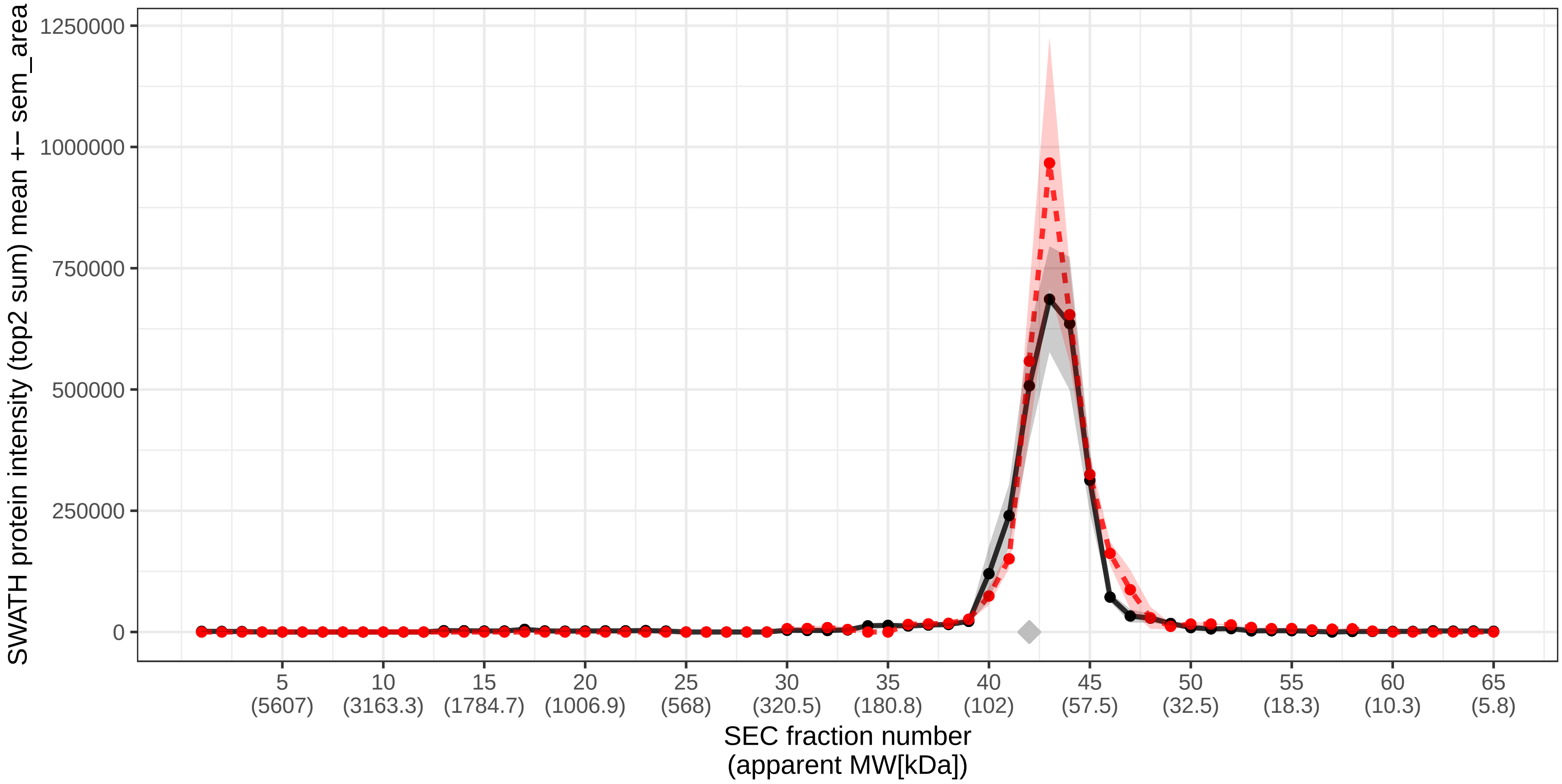

Supplement: Data S1. SEC-SWATH-MS Protein Chromatograms, Related to Figure 1 [file mmc6.zip › SECchrom_P51812_KS6A3_HUMAN_RPS6KA3_ISPK1_MAPKAPK1B_RSK2.pdf]
